# Supplementary material for: Development of Tumor Cell-Based Vaccine with IL-12 Gene Electrotransfer as Adjuvant
Source: Vaccines (Basel). 2020 Mar 2;8(1):111. doi: 10.3390/vaccines8010111 (PMC7157224; doi:10.3390/vaccines8010111)
Supplement: Supplementary file 1 [file vaccines-08-00111-s001.pdf]

vaccines

MDPI

Article

Development of Tumor Cell-Based Vaccine with IL-12 Gene Electrotransfer as Adjuvant

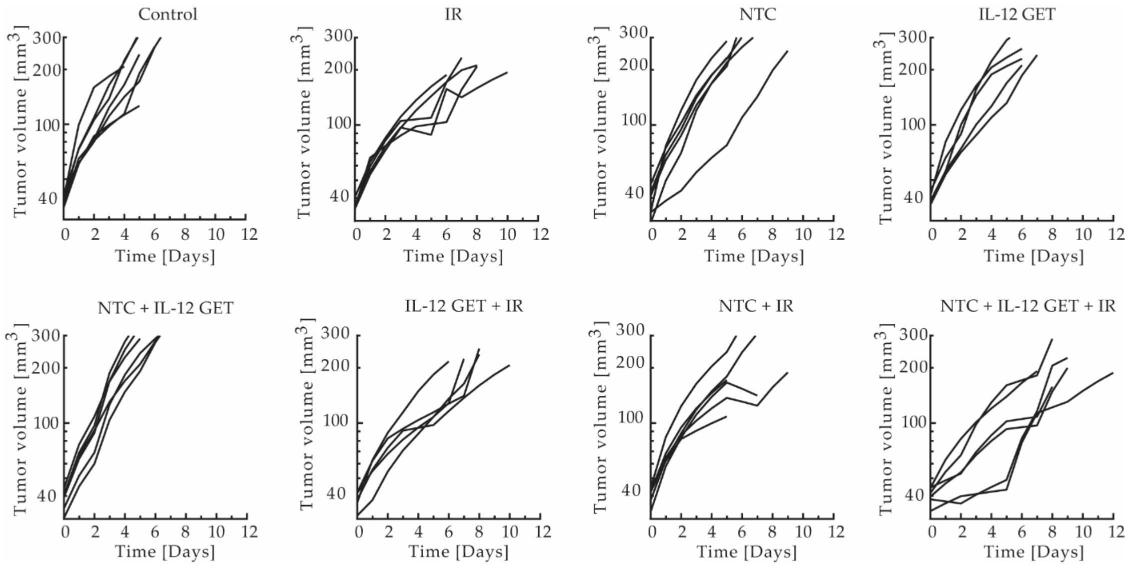

Figure S1. Pilot B16-F10 vaccination: Individual mouse tumor growth curves. NTC = 1 unit of the B16-F10 non-viable tumor cells (0.5mg); GET = gene electrotransfer; IR = local tumor irradiation with 10 Gy.

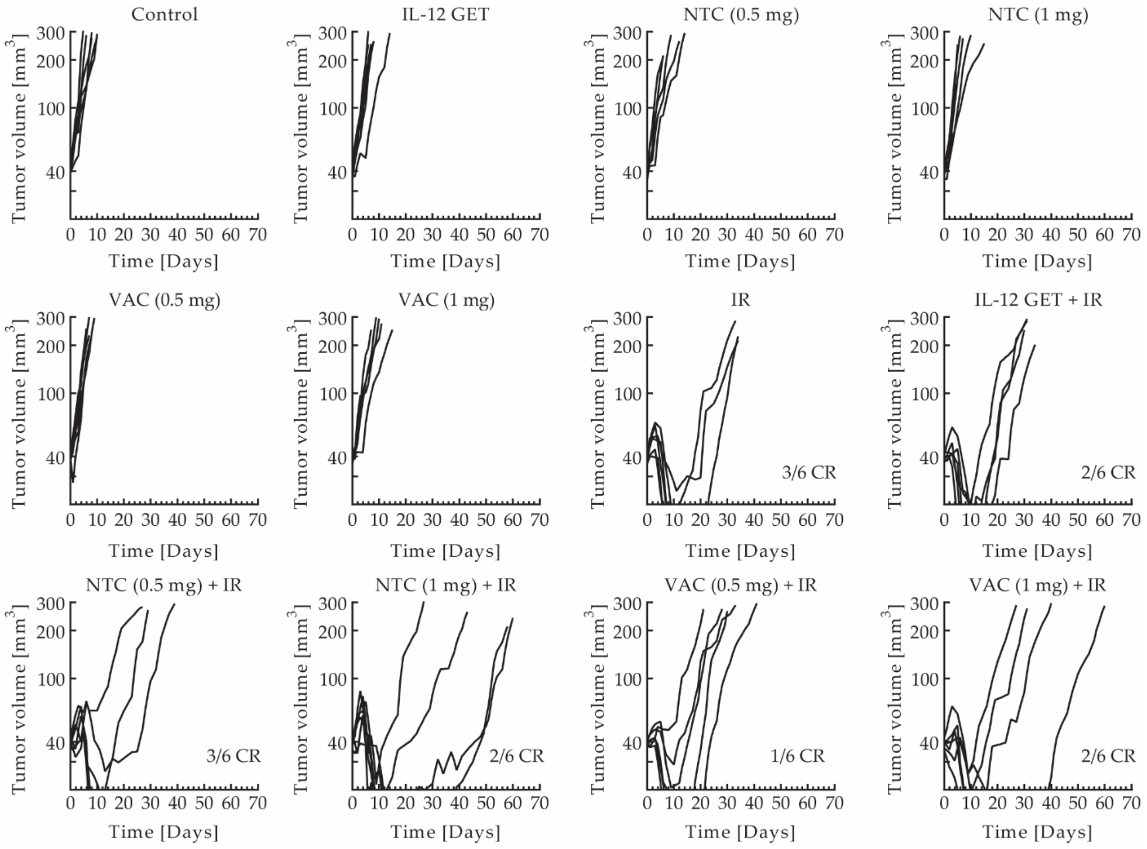

Figure S2. Adjusted B16-F10 vaccination: Individual mouse tumor growth curves. GET = gene electrotransfer; NTC = 1 unit of non-viable B16-F10 tumor cells (0.5 mg or 1 mg), prepared using the

adjusted vaccine preparation protocol; VAC = 1 unit of the B16-F10 vaccine (0.5 mg or 1 mg), prepared using the adjusted vaccine preparation protocol, including IL-12 GET.

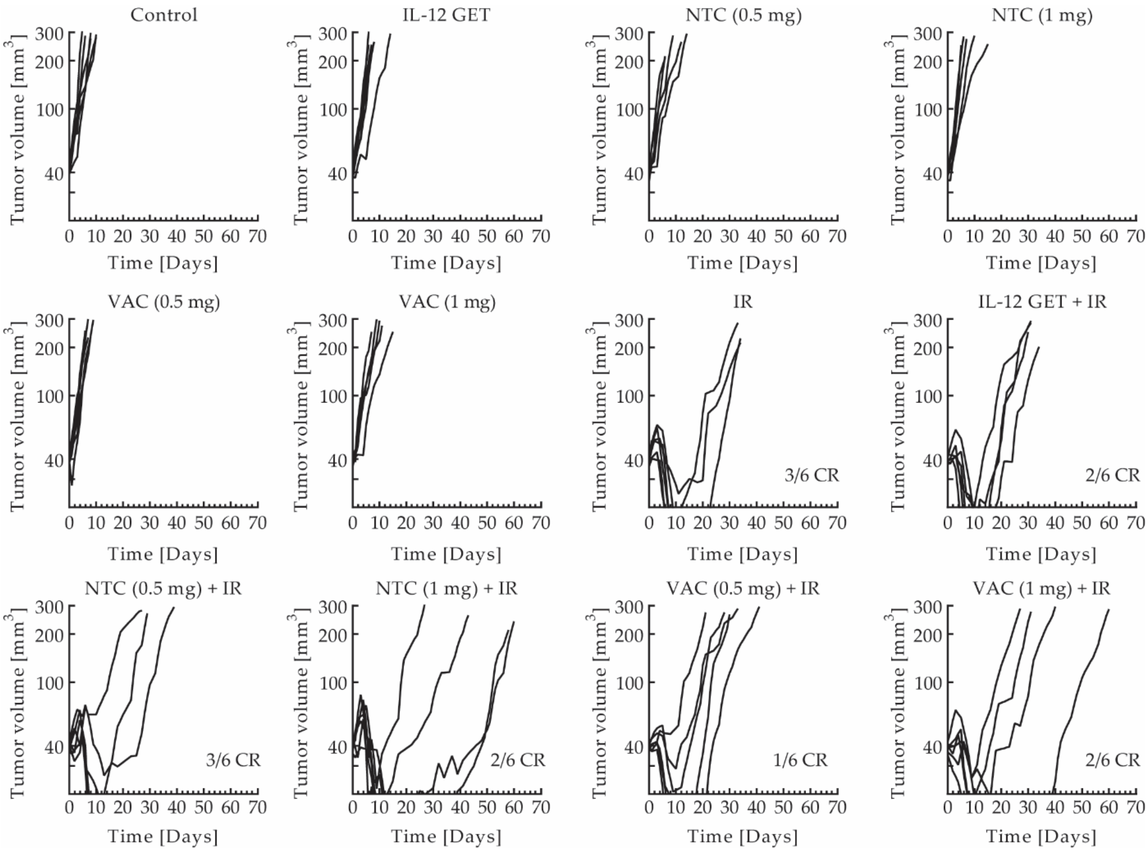

Figure S3. Adjusted CT26 vaccination: Individual mouse tumor growth curves. CR = complete response; GET = gene electrotransfer; NTC = 1 unit of non-viable CT26 tumor cells (0.5 mg or 1 mg), prepared using the adjusted vaccine preparation protocol; VAC = 1 unit of the CT26 vaccine (0.5 mg or 1 mg), prepared using the adjusted vaccine preparation protocol, including IL-12 GET.

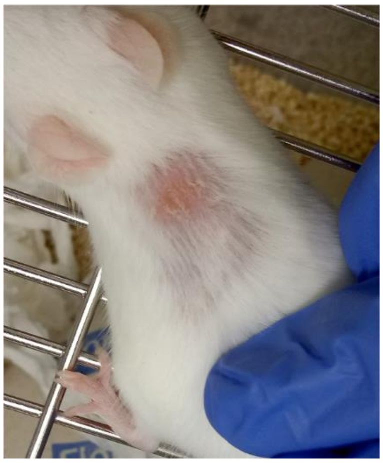

Figure S4. Example of delayed type hypersensitivity-like reaction at the vaccination site.

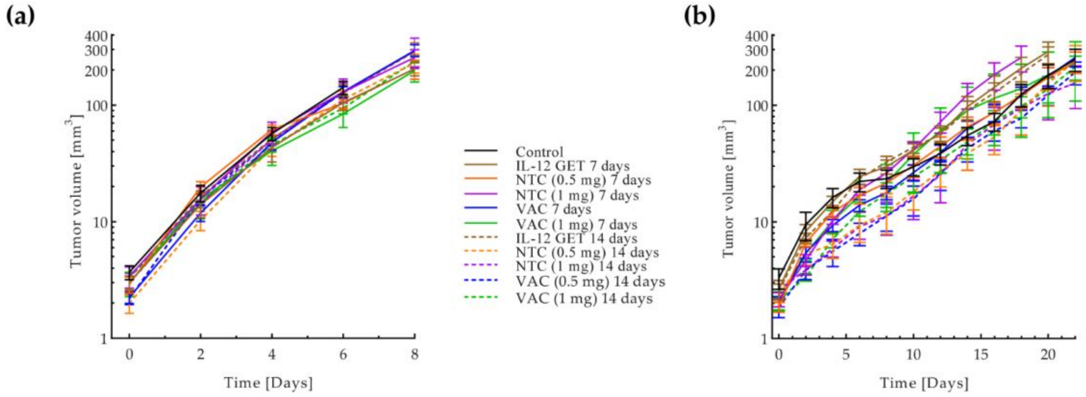

Figure S5. B16-F10 and CT26 preventative vaccination: Tumor growth curves. **(a)** Preventative B16-F10 vaccination - tumor growth curve. Tumor volume in logarithmic scale plotted against time. **(b)** Preventative CT26 vaccination - tumor growth curve. Tumor volume in logarithmic scale plotted against time. Legend applies to both graphs. GET = gene electrotransfer; NTC = 1 unit of non-viable B16-F10 or CT26 tumor cells (0.5 mg or 1 mg), prepared using the adjusted vaccine preparation protocol; VAC = 1 unit of the B16-F10 or CT26 vaccine (0.5 mg or 1 mg), prepared using the adjusted vaccine preparation protocol, including IL-12 GET.

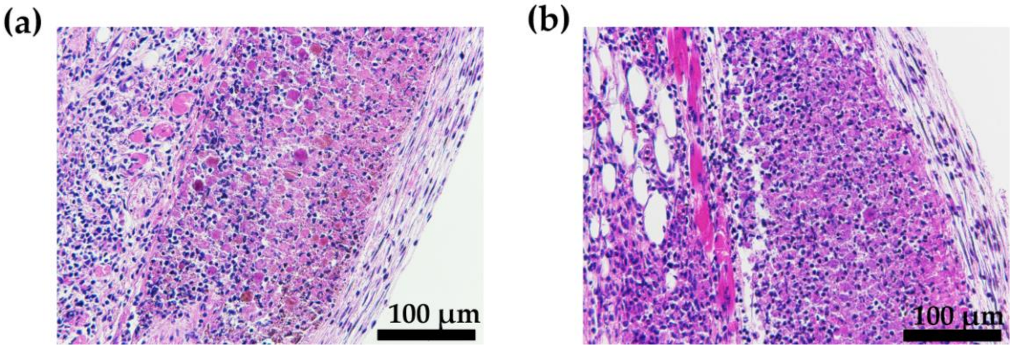

Figure S6. Adjusted B16-F10 and CT26 vaccination: H&E stained vaccination sites. **(a)** The vaccination site in the B16-F10 tumor model. **(b)** The vaccination site of the in the CT26 tumor model. H&E = hematoxylin and eosin staining.

© 2020 by the authors. Submitted for possible open access publication under the terms and conditions of the Creative Commons Attribution (CC BY) license (http://creativecommons.org/licenses/by/4.0/).
